# Anticancer Drug Development

**DOI:** 10.1038/sj.bjc.6600309

**Published:** 2002-05-03

**Authors:** P Loadman

**Affiliations:** Cancer Research Unit, University of Bradford, UK

## Abstract

*British Journal of Cancer* (2002) **86**, 1665–1666. DOI: 10.1038/sj/bjc/6600309 www.bjcancer.com

© 2002 Cancer Research UK

## 

Edited by BC Baguley and DJ Kerr. Publishers: Academic Press. ISBN 0-12-072651-3. £66.95, $99.95.

Due to the tremendous increase in our understanding of the cancer process and cause, development of drugs to selectively interfere with and inhibit tumour growth has now entered a new and exciting era of the target orientated approach. Drugs are now being developed against specific proteins involved in cancer cell growth that have been uncovered over the last 10 years or so. These differences cover all areas of cancer biology, from cell signalling pathways to tumour hypoxia and from angiogenesis to cell death pathways. It therefore follows that to develop drugs that interfere with these complex pathways, a comprehensive knowledge of the cancer process is needed by today's cancer researcher. This book gives a very comprehensive overview of the whole of the anticancer drug development process today, from chemical synthesis to the clinic.

The book begins with a brief chapter on the history of chemotherapy describing the problems of today's anticancer drugs, the lack of specificity or low therapeutic index. Individual sections on specific areas follow, where the emphasis is on uncovering new targets for therapeutic interference. These include the cell cycle, signal transduction pathways and apoptosis. Not only are these detailed and up-to-date reviews of the topic, but highlight areas of possible therapeutic intervention throughout the text, including compounds that are already known to act on these areas, very useful for those not familiar with the field. Each chapter concludes with strategies for future drug development.

Attempts at inhibiting the cancer metastatic process have been high profile recently with the MMP inhibitors, Batimastat and Marimastat, entering the clinic. Cancer metastasis is a complex process, and this chapter not only gives an overview of the subject but also describes in some detail the thinking behind the design of existing and future MMP inhibitors.

Drug resistance pathways have long been an area of interest for therapeutic intervention and these are brought up-to-date with our current understanding of P-glycoprotein, NF-κB and the potential role of tumour physiology in drug resistance. Anti-angiogenic drugs and antivascular agents are not quite the same but have been grouped to cover all aspects of tumour vasculature as a target. Here we see slight overlap with other chapters, and indeed the editors do comment on the overlap between chapters as each chapter is written by independent authors. This overlap is inevitable (but minimal), as tumour vasculature and angiogenesis is bound to overlap with MMP expression and inhibition which is bound to overlap with cell signalling, and so on. The GDEPT concept is dependent on the activation of prodrugs at the tumour site. Several useful enzyme-prodrugs systems are covered here as well as the latest antigenic targets available for ADEPT. These are two concepts that have shown promise preclinically but are still under development in the clinic.

The drug discovery process is still a mixture of random screening and rational drug design. There follows three chemistry based chapters on drug design. Rational drug design and organic synthesis (including combinatorial chemistry) with concluding remarks which give pointers as to the future direction of the field. Natural products of course have had a major role to play in cancer chemotherapy over the years. This chapter is written by chemists who have the enviable job of travelling the world looking for natural products with novel mechanisms of action. Here bryostatin is used as an example to include details of structural modification and *in vitro* evaluation. Other natural products are certain to have their influence on cancer drug development for some time to come.

Some of the technological advances over recent years would have been unimaginable not too long ago. We are now in the era described as post genomic drug discovery. Not only has the technology advanced at an impressive rate but so has the information gained from these technologies. Microarray analysis, genomics and proteomics provide gigantic amounts of data for the cancer researcher. These technologies have now been matched in the drug discovery process by high throughput screening techniques. This chapter brings together the previous 13 by describing the drug screening processes itself, including the roles and values of target identification, target validation and combinatorial chemistry in the drug discovery and development process.

The majority of reviews on drug development were dominated by the NCI screening technologies. It is not really possible to publish a review on anticancer drug development without including the NCI screen, but the chapter on tumour cell cultures in drug development takes a slightly different angle. The NCI screen is covered in some detail but so are useful sections on three-dimensional cultures and drug diffusion assays. Three-dimensional cultures of characterised cell lines take *in vitro* drug screening a step closer to the clinical situation. It is appropriate then to follow this chapter with one on animal screening systems. Based primarily on the experiences of those in Freiburg, who probably have one of the largest animals screening facilities in Europe. With over 350 tumours all characterised for target expression, the emphasis is placed on the characterisation of the tumours and clinical relevance of the tumour models in mice. There is also an overview of these facilities in Freiburg.

Extensive preclinical pharmacological and toxicological studies prior to clinical trial are essential, not only to gain a thorough understanding of the compounds pharmacology but also to aid in the difficult decision of which starting dose to use in the patient. Extrapolation of animal data to humans is discussed in some detail, comparing the relevance of preclinical information from mouse, rat, dog and monkey. Preclinical data from six compounds that have entered the clinic is used as an example, and the authors conclude that the preclinical animal models used are impressive in their prediction of human MTD (maximum tolerated dose) and DLT (dose limiting toxicities).

This detailed preclinical data can then be used to optimize the design of the clinical trials. This account explains in some detail how a clear understanding of a drugs pharmacology can lead to improved clinical use. A greater understanding of these pharmacokinetic-pharmacodynamic relationships has led to optimization of the administration and delivery of new agents in Phase I trials.

A major new development in clinical trial design is the ability to measure the distribution and, to a certain extent, the pharmacodynamic effect of these new agents in a non-invasive manner. PET (positron emission tomography) has a whole chapter dedicated to the technique and it is clear the power of the technique will influence the design of future clinical trials. PET can now be used for pharmacokinetic analysis with examples, for 5-FU and temozolomide given. It can be used for pharmacodynamic studies, for example drug receptor interactions and cellular proliferation, even detecting apoptotic cells *in situ*.

The concluding chapter discusses trials of the newly emerging mechanistic drugs. It describes trial design for the compounds such as copolymers, intended to take advantage of the leaky vasculature of tumours, and signal transduction inhibitors where it is now necessary to build in pharmacodynamic endpoints, showing target inhibition/activation, into clinical trials alongside the currently established pharmacokinetic monitoring.

In summary, this is a really extensive and comprehensive book on the drug development process. It has detailed chapters on potential targets, the drug design process, the drug screening process and the design of future clinical trials to cope with these new mechanistic based drugs. There is very little overlap between chapters and all are written by experts in the field. Hundreds of useful references are included for those wanting to go further. My one and only problem was finding the colour prints tucked away at the back. Maybe they were too expensive to include in the text, but I found them eventually. Congratulations to the editors as this must have been quite a task. Very good value for $100 and essential reading for both the scientist and Ph.D. student. Definitely one for the library to stock.

